# Is isolation by adaptation driving genetic divergence among proximate Dolly Varden char populations?

**DOI:** 10.1002/ece3.1113

**Published:** 2014-05-22

**Authors:** Morgan H Bond, Penelope A Crane, Wesley A Larson, Tom P Quinn

**Affiliations:** 1 School of Aquatic and Fishery Sciences, University of WashingtonBox 355020, Seattle, Washington, 98195; 2 Conservation Genetics Laboratory, U.S. Fish and Wildlife Service1011 East Tudor Road, Anchorage, Alaska, 99503

**Keywords:** Adaptation, landscape genetics, life history, salmon

## Abstract

Numerous studies of population genetics in salmonids and other anadromous fishes have revealed that population structure is generally organized into geographic hierarchies (isolation by distance), but significant structure can exist in proximate populations due to varying selective pressures (isolation by adaptation). In Chignik Lakes, Alaska, anadromous Dolly Varden char (*Salvelinus malma*) spawn in nearly all accessible streams throughout the watershed, including those draining directly to an estuary, Chignik Lagoon, into larger rivers, and into lakes. Collections of Dolly Varden fry from 13 streams throughout the system revealed low levels of population structure among streams emptying into freshwater. However, much stronger genetic differentiation was detected between streams emptying into freshwater and streams flowing directly into estuarine environments. This fine-scale reproductive isolation without any physical barriers to migration is likely driven by differences in selection pressures across freshwater and estuarine environments. Estuary tributaries had fewer larger, older juveniles, suggesting an alternative life history of smolting and migration to the marine environment at a much smaller size than occurs in the other populations. Therefore, genetic data were consistent with a scenario where isolation by adaptation occurs between populations of Dolly Varden in the study system, and ecological data suggest that this isolation may partially be a result of a novel Dolly Varden life history of seawater tolerance at a smaller size than previously recognized.

## Introduction

Genetic population substructuring has been detected in Northern Hemisphere marine and anadromous fishes, often resulting from postglacial colonization (King et al. 2001; Cunningham et al. 2009; Hasselman et al. 2013). An ongoing balance between reproductive isolation resulting from homing to natal breeding areas and gene flow among populations caused by successful reproduction of individuals that stray (reproduce in a nonnatal site) maintains varying levels of structuring broadly observed as isolation by distance (Olsen et al. 2011; Templin et al. 2011; Moran et al. 2012). The origins of populations in different glacial refuges blend with ecological processes over a range of broad and local scales to create the observed contemporary population structure (Churikov and Gharrett 2002; Castric and Bernatchez 2003; Petrou et al. 2013). Adaptation occurs through selection of successful traits within a locally reproducing population, isolated from other populations by geography or homing behavior (Ricker 1972; Taylor 1991; Quinn 2005; Fraser et al. 2011). These processes occur in all species but are most closely studied in salmonids (Salmonidae) as a result of their especially rich adaptation patterns, natal homing fidelity, broad spatial distributions, and need for information on genetic population structure to manage valuable fisheries (Shaklee et al. 1999; Neville et al. 2006; Wood et al. 2008).

Within salmonids, many studies have identified population structure, ranging from broad surveys of genetic diversity (Seeb and Crane 1999; Beacham et al. 2006; Templin et al. 2011) and work on fine-scale divergence of proximate populations (e.g., Adams and Hutchings 2003; Lin et al. 2008), as well as studies related to the status of imperiled stocks or other conservation goals (e.g., Small et al. 2009; Heggenes et al. 2011; Van Doornik et al. 2011). In these cases, genetic data are used to define discrete populations and inform management practices that preserve unique evolutionary lineages (Waples and Gaggiotti 2006).

Recently, studies of population structure have shifted to understanding the environmental and evolutionary processes that lead to breaks in the traditional isolation by distance model (Bradbury and Bentzen 2007; Nosil et al. 2009; Orsini et al. 2013). In some cases, strong genetic separation is maintained for proximate populations of fishes by small scale shifts in environmental conditions, even in the absence of physically isolating barriers (e.g., waterfalls). The underlying mechanism maintaining the population structure in these cases is often environmental gradients that select against fish straying from their natal spawning area, resulting in morphological, physiological, or life-history differences between genetically distinct groups (Hendry et al. 2000; Lin et al. 2008). For example, strong genetic separation between sockeye salmon *Oncorhynchus nerka* spawning in creeks and adjacent beaches, driven largely by the selective pressures on body size and shape in each habitat (Hendry et al. 2000; Lin et al. 2008). Understanding the environmental gradients that create and maintain finescale isolation among populations (Bradbury et al. 2013) is an important and underappreciated component of fish conservation, as the added diversity may increase the overall resilience of a species regionally (Hilborn et al. 2003; Greene et al. 2009; Schindler et al. 2010).

**Figure 1:**
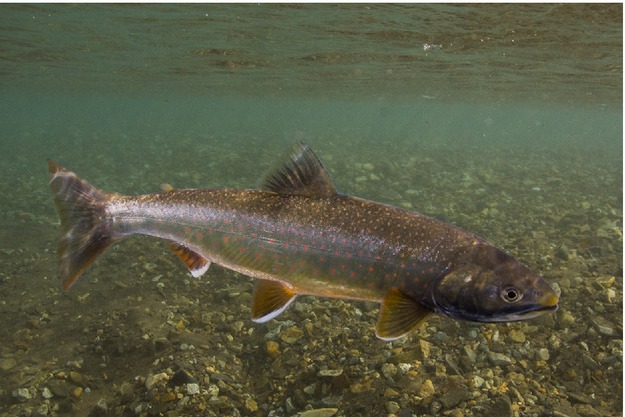
Adult Dolly Varden in spawning coloration observed near Disappearing Creek, a tributary of Chignik Lake (Photo credit: M. H. Bond).

In nonanadromous salmonids, populations often show remarkable genetic structure, resulting from allopatric divergence during glaciation, significant changes to watershed structure during de-glaciation, or low postglacial connectivity due to the advent of barriers, (Latterell et al. 2003; Whiteley et al. 2006, 2010). Conversely, the high dispersal of marine fishes often leads to levels of genetic structure that are nonexistent or near lower detection limits (Gyllensten 1985; Ward et al. 1994; Waples 1998). Anadromous salmonids often fall somewhere between, where high levels of homing lead to population structure, but this structure may be decreased by strays that spawn outside of their natal stream. In iteroparous, facultatively anadromous fishes (i.e., those where marine migrations are possible but not obligatory) like Dolly Varden char (*S. malma,* Fig. 1), populations fall on a continuum between almost exclusively nonanadromous (Palmer and King 2005) to predominantly anadromous (Armstrong and Morrow 1980; Maekawa and Nakano 2002). Dolly Varden are philopatric, and several studies have shown significant population structure among streams or watersheds (Everett et al. 1997; Rhydderch 2001; Crane et al. 2005a). However, fewer studies have shown genetic population structure on small spatial scales (within watersheds, among reaches, within reaches) in readily connected habitats (but see Currens et al. 2003; Ostberg et al. 2009). Observing population structure in Dolly Varden is complicated by their movement between watersheds at some periods in their lives. For example, in southeastern Alaska, juveniles often rear in natal streams for several years, then may move through marine waters and over-winter in nonnatal watersheds with lakes or other favorable conditions not present in their natal streams (Armstrong 1974, 1984; Bernard et al. 1995). Following extended residence in nonnatal watersheds, individuals will migrate back to their natal site for reproduction (Armstrong 1974, 1984). The availability of overwintering habitat (e.g., lakes, deep rivers, or ice free habitat) is thought to drive much of the observed movement; the suitability of watersheds changes seasonally, and fish move to take advantage of alternate habitats. Therefore, inferences of population genetic structure may be biased if Dolly Varden are sampled when populations are likely to be mixed (Crane et al. 2005b).

**Figure 2:**
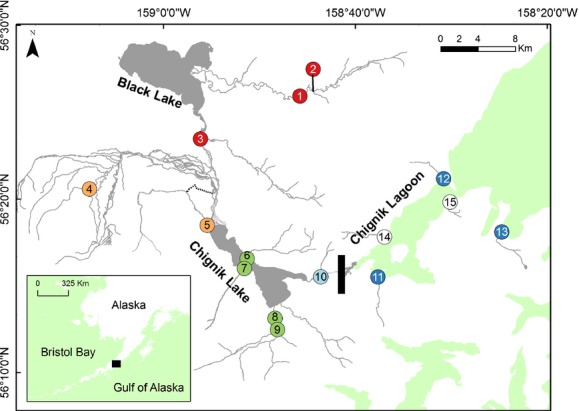
Map of sampling locations with the strongest barrier to gene flow drawn as a black line between populations 10 and 11. The barrier was identified with the program BARRIER 2.2 (Manni et al. 2004). Point shape indicate clusters defined from PCoA: red; Black Lake, orange; West Fork River, green; Chignik Lake, light blue; Chignik River, dark blue; Estuary, white; Estuary (fish length measurement only). The dashed stream channel near number 5 indicates the former (ca. 1963) streambed of Bearskin Creek. See Table 1 for additional details about each sampling location by point number.

The Chignik Lakes system on the Alaska Peninsula (Fig. 2) presents anadromous fishes with several distinct spawning and rearing habitats in close proximity, including headwater streams, larger rivers, two different lakes within the drainage basin itself, and small streams which drain directly into brackish or fully marine lagoon waters nearby. These features make this an excellent system for studying population structure as there are no physical barriers to movement among the heterogeneous breeding habitats. Significant genetic population structure has been demonstrated for sockeye salmon within the Chignik system, including differentiation between spawning locations, ecotypes, and spawn timing (Creelman et al. 2011). Further, several morphs of pygmy whitefish, *Prosopium coulterii* (McCart 1970; Gowell et al. 2012), and threespine stickleback, *Gasterosteus aculeatus* (Narver 1969), exist within the Chignik watershed, indicating sufficient environmental variation for phenotypic plasticity or evolutionary processes to create multiple morphs in near sympatry. Initial genetic research identified Dolly Varden as the only char species within the drainage and suggested fine-scale population structure (Taylor et al. 2008). This genetic structure may be influenced in large part by selection on life-history traits among juvenile rearing habitats. For example, tributaries of the lagoon are generally small with especially low flows during the summer and winter periods; Dolly Varden fry spawned in these habitats likely must move into marine waters during their first year of life to find more suitable rearing and overwintering habitats, so early anadromy may be favored. Conversely, the Alec River, near the upper end of the watershed, is deep and flows throughout winter months. Fry spawned in tributaries of the Alec River need only move a short distance to find suitable overwintering habitat in the river or lake and may never initiate anadromous migrations (Bond 2013). Because the traits associated with anadromous migrations (Quinn et al. 2002) and the timing of smolt transformation are heritable and the selection on these traits likely differs among habitat types within the watershed, strong selection against strays may promote reproductive isolation.

Here, we used genetic data to assess population structure among collections of Dolly Varden made throughout the Chignik Lakes watershed in areas accessible to anadromous fishes (i.e., below any barriers to migration). Patterns of population structure for streams draining into freshwater were relatively subtle and generally mirrored those of sockeye salmon in this system (Creelman et al. 2011). However, greater genetic differentiation was observed between proximate populations spawning in streams that drain into freshwater and streams that drain to estuarine environments. We suggest that this reproductive isolation is caused by variable selective pressures across these two environments and support our conclusions with juvenile size distribution data across spawning habitats.

## Methods

### Study site

The 1536 km^2^ Chignik watershed is composed of two connected lakes that drain into a large brackish lagoon with a connection to the ocean (Westley et al. 2008; Simmons et al. 2013; Fig. 2). Connectivity among habitats is largely through two low-gradient rivers that connect Chignik Lagoon to Chignik Lake, and Chignik Lake to Black Lake (10 m elevation). At least 12 fish species inhabit the freshwater portions of the watershed, but Dolly Varden are the only large bodied resident fish (the other large fishes being semelparous Pacific salmon that do not feed in freshwater as adults). Apart from a commercial and subsistence fishery on sockeye salmon, the watershed is nearly free of anthropogenic disturbance, and exploitation of nonsalmon species is low. Dolly Varden within the watershed are abundant and are found at some life stage in nearly every stream in the watershed (M. H. Bond, unpublished data), including the following: those that drain directly into saltwater habitats, tributaries of larger rivers, and tributaries of the two lakes, which markedly differ in depth, thermal regime, productivity, and other attributes (Westley et al. 2010; Griffiths et al. 2011). In addition, Chignik Lakes Dolly Varden express a wide range of migratory phenotypes, from fully resident forms, to individuals smolting at a variety of ages (Bond 2013).

### Sample collection

Assessments of genetic structure in fishes typically involve sampling spawning individuals. However, the fall upstream migration of both mature and immature individuals combined with potentially low rates of philopatry among nonspawners may obscure real population structure among spawning locations. Juveniles are not normally used in population structure studies when spawning adults are readily available because of the potential for sampling family groups or failing to capture a spatially representative sample, which could yield biased results (Allendorf and Phelps 1981; Hansen et al. 1997; Banks et al. 2000). However, because adults were in marine waters during the summer sampling period, we sampled fry, which should provide a representative sample of spawners for a given collection site as long as some precautions are taken (Garant et al. 2000).

Tissue samples were collected from Dolly Varden fry (young of the year, as inferred from length-frequency distributions) in streams throughout the Chignik watershed, and one from an adjacent watershed (Table 1, Fig. 2) using single-pass electrofishing in June and July of 2009 and 2010. Upon collection, whole individuals or fin clips were preserved in 95% ethanol following euthanasia in buffered methane sulfonate (MS-222). Although all sampling was conducted in streams, sites in streams draining directly to the lagoon or ocean are collectively referred to as Estuary streams, whereas sites in streams that drain to Chignik River or one of the Chignik Lakes are referred to as Freshwater streams. To minimize the likelihood of sampling a family group and to ensure relatively equal sampling across each sampling site, only one Dolly Varden was collected per pool, unless the individuals differed in length by more than 3 mm, in which case two were sampled. No fish greater than 70 mm were sampled to ensure that collected fish were young of the year and unlikely to have originated from another location (Bond 2013). Additionally, in a set of smaller streams where sampling of the entire stream width was possible, we measured the fork length of all Dolly Varden encountered during sampling to determine whether similar size classes of fish were present in both Freshwater and Estuary streams. For comparative purposes, fish length measurements were also made in several Estuary tributaries not included in the genetic analyses.

**Table 1:** Population number, collection location, geographic region, latitude and longitude of creek mouth, number of full sib groups, number of full sib groups per 100 fish sampled, sample size, years of sampling, observed heterozygosity (H_O_), expected heterozygosity (H_E_) and allelic richness (AR) for each population in the study. *Pop no*. corresponds to the population number in Figure 1, *geographic group* is the group used for AMOVA analysis, *N sib groups* is the number of full sibling groups found in each population, *N before* is the number of individuals that were successfully genotyped for each population and *N after* is the adjusted sample size after removing siblings

Pop no.	Population	Abb reviation	Geographic group	Latitude (N)	Longitute (W)	*N* sib groups	Groups/100 fish	*N* before	*N* after	Year	H_O_	H_E_	AR
1	Boulevard Cr.	Boul	Black Lake	56.435267	−158.754067	2	2.0	100	97	2009, 2010	0.77	0.78	11.51
2	Alec Tributary 2 Cr.	Alec	Black Lake	56.432133	−158.763233	1	1.9	52	51	2009	0.76	0.76	11.32
3	Chiaktuak Cr.	Chia	Black Lake	56.391300	−158.936017	4	4.0	101	94	2009, 2010	0.80	0.79	11.89
4	Cloud Cr.	Cloud	Westfork	56.343150	−159.128050	2	4.1	49	46	2010	0.67	0.69	10.80
5	Bearskin Cr.	Bskin	Westfork	56.308483	−158.924300	1	1.1	91	90	2010	0.70	0.70	10.75
6	Cucumber Cr.	Cucu	Chignik Lake	56.276300	−158.855400	9	10.0	90	80	2009	0.79	0.80	11.83
7	Hatchery Pointer.	Hate	Chignik Lake	56.266917	−158.860383	1	1.8	55	54	2009	0.72	0.76	11.17
8	Fonz Cr.	Fonz	Chignik Lake	56.218933	−158.807550	12	11.4	105	73	2009, 2010	0.71	0.71	11.43
9	Disappearing Cr.	Disa	Chignik Lake	56.207967	−158.803417	14	17.7	79	56	2009, 2010	0.74	0.77	10.53
10	Bear Cr.	Bear	Chignik River	56.258650	−158.728333	6	12.0	50	35	2010	0.73	0.72	11.41
11	Metrofania Cr.	Metro	Estuary	56.258233	−158.629783	6	11.8	51	29	2010	0.72	0.73	10.02
12	Spit Cr.	Spit	Estuary	56.352083	−158.514917	11	15.1	73	44	2010	0.71	0.68	10.26
13	Indian Cr.	Indi	Estuary	56.300667	−158.415250	13	13.3	98	68	2010	0.63	0.64	8.58
14	Hume Point Cr.		Estuary	56.296550	−158.617880	Sampled for fish length only							
15	Waterfall Cr.		Estuary	56.329683	−158.504070	Sampled for fish length only							

To compare size distributions of fish from Freshwater and Estuary streams, we compared the mean fork length of fish from each habitat with a Wilcoxon signed rank test in the statistical software package R (R Development Core Team 2011). To determine the relative incidence of older age classes of fish, we used a *z*-test to compare the proportion of fish in each habitat that were larger than 80 mm FL and 115 mm FL, which are minimum size estimates of age 1+ and 2+ fish, respectively, from previous otolith analysis of Chignik Lakes Dolly Varden (Bond 2013).

### Laboratory analysis

Genomic DNA was extracted using a DNeasy 96 Tissue Kit (Qiagen, Valencia, CA), and genetic variation was assessed at 11 microsatellite loci developed from *Oncorhynchus gorbuscha*, *Salvelinus confluentus*, *Salvelinus fontinalis,* and *S. malma* (Table 2). Polymerase chain reactions were conducted in 10 *μ*L reaction volumes comprising 30–50 ng DNA, 1.5–2.5 mmol/L MgCl_2_, 0.8–1 mmol/L dNTPs, 0.1–0.6 *μ*mol/L labeled forward primer, and 0.1–0.6 *μ*mol/L unlabeled reverse primer, and 0.025–0.05 U/*μ*l *Taq* polymerase using a Bio-Rad DNA Engine Tetrad 2 thermocycler set to one cycle of 2 min at 92°C, 30 cycles of 15 sec at 92°C, 15 sec at 55–60°C, and 30 sec at 72° with a final extension for 10 min at 72°C. PCR amplicons were size-separated on an Applied Biosystems 3730 Genetic Analyzer and scored with the program Genemapper version 4.1 (Life Technologies, Grand Island, NY). Genotypes were scored independently by two researchers. Genotypes were compared, and fish with discrepancies were reamplified and rescored until discrepancies were resolved. Two quality control measures were employed. First, PCR amplifications were repeated for 8% of the samples, size-separated, and rescored to check and correct for laboratory errors. In addition, DNA was extracted a second time from approximately every 25th sample collected and genotyped to quantify laboratory error rates. Individuals with >2 missing genotypes were removed from further analyses.

**Table 2:** Information for each locus analyzed in this study including number of alleles (A), allelic richness (AR), observed heterozygosity (H_O_), expected heterozygosity (H_E_), *F*_ST_, *F*_IS_, source of locus, annealing temperature in °C, and the number of cycles used in the PCR amplification

Locus	A	AR	H_O_	H_E_	*F*_ST_	*F*_IS_	Source	T(°C)
OgolA	8	5.617	0.603	0.615	0.057	0.004	Olsen et al. (1998)	55
Sco202	18	13.412	0.881	0.881	0.051	−0.004	DeHaan and Ardren (2005)	60
Sco204	42	25.435	0.882	0.919	0.016	0.000	DeHaan and Ardren (2005)	60
Sfol8	2	1.392	0.118	0.112	0.026	−0.033	Angers et al. (1995)	55
Smm21	15	7.258	0.500	0.533	0.051	0.011	Crane et al. (2004)	55
Smm22	26	17.345	0.912	0.881	0.012	−0.001	Crane et al. (2004)	55
Smm24	38	24.597	0.750	0.836	0.018	0.010	Crane et al. (2004)	55
Smm3	6	5.355	0.750	0.705	0.079	0.011	Crane et al. (2004)	58
Smm41	33	19.683	0.838	0.920	0.013	0.037	USFWS, unpublished	58
Sm m44	28	11.047	0.279	0.269	0.035	−0.014	USFWS, unpublished	55
Smm5	6	3.436	0.382	0.356	0.094	0.046	Crane et al. (2004)	55

### Sibling detection

We used the maximum likelihood method implemented in the program COLONY 2.0.3.5 (Wang 2004) to identify potential sibling groups in our data. COLONY was run twice for each collection with the following parameters: mating system – female and male polygamy with no inbreeding, species – diecious, length of run – medium, analysis method – Fl-PLS combined, no updating of allele frequencies. Full sibling groups that were identified in either run were denoted as sibships, and one individual from each sibling group with the least amount of missing data was retained for further analyses.

### Statistical analyses

After removing siblings, collections taken from the same location across multiple years were pooled in accordance with Waples (1990). The program ML-NULLFREQ (http://www.montana.edu/kalinowski/) was then used to test for the presence of null alleles. Exact tests for deviations from Hardy–Weinberg and linkage equilibrium were conducted for each locus in the program GENEPOP 4 (Rousset 2008). The initial significance level for these tests was 0.05, and we applied a sequential Bonferroni correction (Rice 1989) to correct for multiple tests. Allelic richness (AR), number of alleles (A), and observed and expected heterozygosities for each locus and each population were calculated in FSTAT 2.9.3 (Goudet 1995) and ARLEQUIN 3.5 (Excoffier and Lischer 2010). Additionally, we tested for significant differences in allelic richness and observed heterozygosity between Freshwater and Estuary streams with permutation tests in FSTAT (1000 permutations, significance level = 0.05). Locus specific values of *F*_ST_ and *F*_IS_ (Weir and Cockerham 1984) were calculated in GENEPOP.

Genetic differentiation among collections was estimated across all loci with pairwise *F*_ST_ values (Weir and Cockerham 1984) calculated in GENEPOP. We then conducted principal coordinate analysis (PCoA) using pairwise *F*_ST_ values in GenAlEx (Peakall and Smouse 2012) to visualize the population structure in our data. Two separate PCoAs were constructed to ensure all major patterns of population structure were adequately evaluated: (1) all collections, and (2) only the Freshwater collections. Exact tests of genetic differentiation between collections were conducted in ARLEQUIN 3.5 with 1000 permutations and an initial significance level of 0.01. The sequential Bonferroni method was then used to correct for multiple tests. In addition, genetic relationships among populations were visualized with a neighbor-joining tree based on Nei's *D*_A_ distance (Nei et al. 1983) constructed in the program POPTREE2 (Takezaki et al. 2010). We conducted 10,000 bootstraps to determine the support for each node.

We further used the Bayesian MCMC approach implemented in STRUCTURE 2.3.4 (Pritchard et al. 2000) to infer the number of major genetic clusters in our data. This program groups individuals into *K* genetic clusters by minimizing overall deviation from Hardy–Weinberg and linkage equilibrium within clusters. STRUCTURE analysis was conducted with the default model parameters with one exception. We used sampling locations as a prior as suggested by Hubisz et al. (2009). This approach produces more accurate results than a model where sampling locations are not used as priors and does not appear to create artificial structure in data sets with weak structure (Hubisz et al. 2009). STRUCTURE was run separately for the entire data set and a Freshwater data set. For each data set, ten trials were conducted for predefined *K* values from one to ten. Each trial consisted of a burn-in period of 100,000 iterations followed by 500,000 iterations. The most likely value of *K* for each data set was evaluated with raw probability values of LnP (*X*|*K*) given by the program and the Δ*K* method (Evanno et al. 2005), and the results were visualized with STRUCTURE HARVESTER (Earl and vonholdt 2012). If the raw probability and Δ*K* methods indicated a different number of clusters, the results based on the Δ*K* method were adopted as suggested by Evanno et al. (2005). We conducted an analysis of molecular variance (AMOVA) in ARLEQUIN 3.5 to examine the level of variation within and among groups based on sample sites. The hierarchy for this analysis was chosen based on the geography of the Chignik system and the clustering patterns from the PCoAs and STRUCTURE analysis: (1) Black Lake; (2) West Fork; (3) Chignik Lake; (4) Chignik River; and (5) Estuary (Table 1). Separate AMOVAs were conducted for (1) the entire data set; and (2) only the Freshwater collections.

Simple and partial Mantel tests with 1000 randomizations were used to test for relationships between genetic differentiation (pairwise *F*_ST_), geographic distance, and salinity. Geographic distance was the shortest waterway distance between the stream mouths of each pair of collections and was estimated by hand using ARCGIS 10 (ESRI, Inc., Redlands, CA, USA). Mean values for salinity in ‰ for each Estuary stream were obtained from lagoon surface water near stream inlets every 10 days throughout the sampling period.

We used the program BARRIER 2.2 (Manni et al. 2004) to identify the most pronounced barriers to gene flow in the Chignik system. BARRIER takes a pairwise matrix of genetic (*F*_ST_) and geographic distances then implements the Monmonier's maximum difference algorithm (Monmonier 1973) to identify genetic barriers in the data set. The robustness of each barrier was assessed by bootstrapping over loci to generate 100 matrices of genetic differentiation then tabulating the number of bootstraps that supported the barrier (cf. Olsen et al. 2011).

## Results

### Sample collection and laboratory analysis

Genetic samples were obtained from 1017 fry representing 13 collections in the Chignik system (Table 1), 10 in Freshwater streams and three in Estuary streams. Of these, 994 (97.7%) were successfully genotyped. DNA reextraction and analysis of 40 individuals yielded four miscalls of 860 replicated alleles. Most sampled fry were small (mean fork length = 32.6 mm, SD = 6.2 mm) and likely captured soon after emergence. In the set of smaller streams where assessment of all fish present in the sampled section was feasible, we calculated the average fork length of Dolly Varden, as well as size frequency distributions for Freshwater and Estuary streams (Table 3). Freshwater streams (mean = 54.8 mm, SD = 32.8) had larger fish than Estuary streams (mean = 42.8 mm, SD = 20.3 *W*_(1079)_ = 162595.5, *Z* = 4.78, *P* < 0.001. In addition, significantly more large individuals (>80 mm FL) were found in Freshwater streams (16.76%), compared to Estuary streams (7.14%, *z* = 4.57, *P* < 0.001). Similarly, no individuals >115 mm fork length were found in Estuary streams, indicating few larger, older individuals in Estuary stream habitat compared to Freshwater streams where they comprised 8.6% of the fish encountered.

**Table 3:** Average fork length of Dolly Varden captured in Freshwater and Estuary tributaries, ±SD. In addition, the percentage of fish captured in Freshwater and Estuary tributaries that were greater than 80-mm fork length are indicated and were significantly different (*Z* = 4.57, *P* < 0.001). Hume Cr. and Waterfall Cr. are sampled for length only

Site	*n*	Mean fork length (±SD)	% ≥80 mm FL	% ≥115 mm FL
Freshwater	662	54.8 (±32.8)	16.8	8.6
Alec Trib l Cr.	20	80.8 (±22.3)		
Bear Cr.	156	82.7 (±45.2)		
Cucumber Cr.	157	43.2 (±23.1)		
Disappearing Cr.	87	37.8 (±10.5)		
Fonz Cr.	129	43.3 (±16.3)		
Hatchery Cr.	68	62.3 (±20.2)		
Cloud Cr.	45	41.6 (±22.1)		
Estuary	419	42.8 (±20.3)	7.14	0
Hume Cr.	53	51.9 (±23.6)		
Indian Cr.	128	37.7 (±21.4)		
Metrofania Cr.	113	44.6 (±16.1)		
Spit Cr.	90	45.2 (±20.5)		
Waterfall Cr.	35	36.1 (±15.1)		

### Sibling detection

Sibship analysis in COLONY revealed 312 putative full siblings partitioned across 82 full sibling groups ranging from 2 to 18 individuals (average = 2.9 individuals, Table 1). Concordance between COLONY runs was over 95%, and conflicts were generally from full sibling groups of two individuals that were found in one run but not in the other. The number of sibling groups per population varied (1–14, average = 6.3). In general, collections from Black Lake and West Fork appeared to contain fewer sibling groups than populations from Chignik Lake, Chignik River and Chignik Lagoon.

### Statistical analyses

Sample sizes across the 13 populations ranged from 29 to 97 (average = 63.8) after removing siblings and pooling collections from multiple years (Table 1). Sample sizes for some populations were small, but simulations suggest that sample sizes of 25–30 per population are sufficient to accurately estimate allele frequencies from typical microsatellite data (Hale et al. 2012). Analysis with ML-NULLFREQ did not reveal any potential null alleles, therefore it was unnecessary to estimate allele frequencies at null alleles. Tests for locus-specific deviations from Hardy–Weinberg and linkage equilibrium revealed zero loci that were out of equilibrium in greater than three of the 13 populations. We therefore proceeded with all 11 loci. Levels of observed heterozygosity for each population ranged from 0.63 to 0.80 with an average of 0.71 and allelic richness ranged from 8.58 to 11.89 with an average of 10.60 (Table 1). The Indian Creek collection displayed the lowest levels of heterozygosity and allelic richness and the Chiaktuak Creek collection displayed the highest values for these parameters. Freshwater populations displayed greater allelic richness than Estuary populations (*P* = 0.005; Table 1), but the difference in heterozygosity between these two groups was not significant (*P* = 0.055; Table 1).

**Figure 3:**
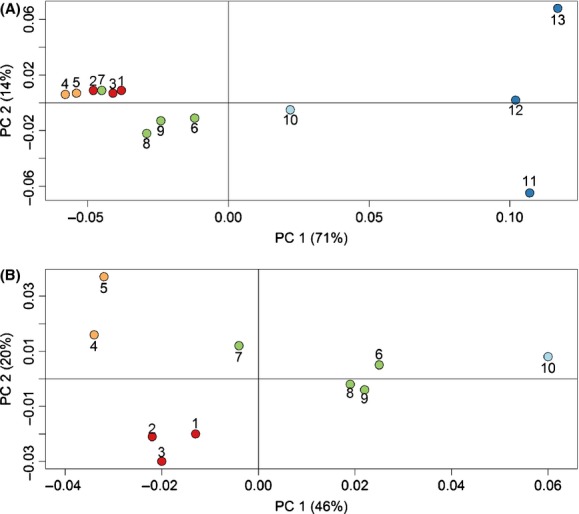
Principal coordinate analysis based on pairwise-*F*_ST_ values for (A) all 13 populations sampled in the Chignik system, and (B) only freshwater populations. Populations are coded by clustering region in both panels: red; Black Lake, orange; West Fork River, green; Chignik Lake, light blue; Chignik River, dark blue; Chignik Lagoon. See Table 1 for additional details about each sampling location by point number.

**Figure 4:**
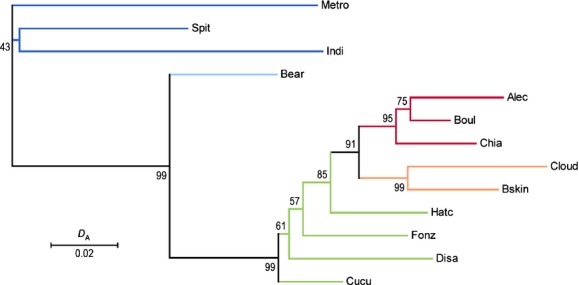
Neighbor-joining tree based on *D*_A_ distance for 13 populations and 11 microsatellites. Percentage bootstrap support is given. Colors correspond to the genetic groups found in Figures 2 and 3.

Principal coordinate analysis revealed that largest genetic differentiation in the Chignik system occurred between the Estuary and Freshwater collections (PC1, 71% of variation, Fig. 3A). The Estuary collections were also highly differentiated from each other (PC2, 14% of variation, Fig. 3A). Freshwater collections were generally differentiated according to geography; populations from Chignik River, Chignik Lake, West Fork, and Black Lake formed discrete clusters (Fig. 3B). Despite this general pattern, the Hatchery Beach population clustered between the Chignik Lake and West Fork collections even though it flows into the middle of Chignik Lake. The overall *F*_ST_ across the entire data set was 0.039 and pairwise *F*_ST_ values ranged from −0.001 for the Boulevard Creek – Alec Tributary 1 comparison, to 0.125 for the Cloud Creek – Indian Creek comparison (average pairwise *F*_ST_ = 0.040, Table 4). Genetic differentiation was significant for all but seven population comparisons, and nonsignificant comparisons generally occurred between proximate populations with the exception of the Alec River – Hatchery Beach and Cloud River – Hatchery Beach comparisons (Table 4, Appendix A2, Table 7). In addition, the neighbor-joining tree of Nei's *D*_A_ (Fig. 4) supported the structure found with PCoA analysis.

**Table 4:** Pairwise *F*_ST_ values for each population. Comparisons in bold are significantly differentiated according to exact tests of genetic differentiation conducted in ARLEQUIN 3.5. The significance level for each test was obtained using an initial significance level of 0.01 and the sequential Bonferroni method to correct for multiple tests. See Table 7 for raw *P*-values. Overall *F*_ST_ for the data set is 0.039

	Boul	Alec	Chia	Cloud	Bskin	Cucu	Hate	Fonz	Disa	Bear	Metro	Spit
Alec	−0.001											
Chia	**0.004**	0.002										
Cloud	**0.009**	0.005	**0.009**									
Bskin	**0.011**	**0.012**	**0.016**	0.005								
Cucu	**0.008**	**0.010**	**0.013**	**0.012**	**0.016**							
Hate	**0.008**	0.006	**0.011**	0.006	**0.011**	**0.008**						
Fonz	**0.012**	**0.013**	**0.012**	**0.017**	**0.018**	**0.006**	**0.009**					
Disa	**0.011**	**0.011**	**0.014**	**0.017**	**0.019**	0.004	**0.009**	**0.009**				
Bear	**0.020**	**0.024**	**0.027**	**0.030**	**0.031**	**0.009**	**0.019**	**0.017**	**0.016**			
Metro	**0.095**	**0.106**	**0.101**	**0.115**	**0.114**	**0.064**	**0.106**	**0.080**	**0.078**	**0.045**		
Spit	**0.077**	**0.085**	**0.080**	**0.097**	**0.099**	**0.055**	**0.085**	**0.074**	**0.071**	**0.035**	**0.030**	
Indi	**0.101**	**0.112**	**0.108**	**0.125**	**0.122**	**0.082**	**0.108**	**0.107**	**0.097**	**0.056**	**0.064**	**0.032**

**Figure 5:**
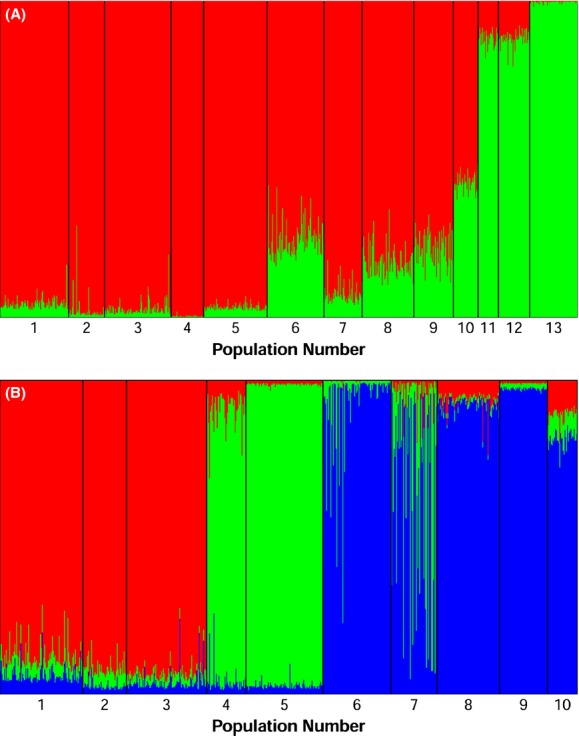
Results from STRUCTURE clustering analysis for two data sets (A) all 13 populations sampled in the Chignik system (*K* = 2), and (B) only freshwater populations (*K* = 3). Results are plotted for the *K* value with the highest probability. Populations are ordered to reflect the geography of the Chignik system and population numbers are found in Table 1.

Clustering analysis revealed similar patterns of population structure as the PCoAs and neighbor-joining tree, and suggested a model of *K* = 2 clusters for the full data set (Fig. 5A) and *K* = 3 clusters for the Freshwater data set (Fig. 5B). Additionally, the three clusters identified in the Freshwater data set generally corresponded to geographic groupings identified in Figure 3B: (i) Black Lake, (ii) Westfork, and (iii) Chignik Lake. Chignik River, however, did not form a discrete cluster despite the fact that it appeared to be highly differentiated based on *F*_ST_ values. Clustering analysis also revealed possible admixture in Cucumber Creek and Hatchery Point Creek collections.

The most likely number of clusters based on raw probability values and the Δ*K* method was the same for the Freshwater data set, but each method suggested a different number of clusters for the full data set. The Δ*K* method suggested *K* = 2 whereas the raw probability values suggested *K* = 5. The Δ*K* method generally appears to produce more robust results than evaluating raw probability values (Evanno et al. 2005); therefore, we focus our discussion on the barplot with *K* = 2 (Fig. 5). We did, however, include a barplot of admixture proportions for *K* = 5 (Appendix A1, 6Figure ). It is important to note that the model with *K* = 5 was able to differentiate Chignik River from the rest of the populations which was not possible in either *K* = 2 including all populations, or *K* = 3 including only Freshwater collections. The probability of each *K* for each data set are shown in Appendix A2, Tables 8 and C.

Hierarchical AMOVAs for the complete data set and for the Freshwater data set both displayed larger variation among groups than within groups (Table 5). Additionally, the amount of variation among groups was much larger when the Estuary collections were included. Simple Mantel tests revealed a significant correlation between genetic and geographic distance, and genetic distance and salinity at the stream mouth, for the complete data set (Table 6). No significant correlation between genetic and geographic distance was found for a data set including only the Freshwater collections. Partial Mantel tests conducted on the complete data set displayed a significant correlation between genetic distance and salinity when corrected for geographic distance but did not display a significant correlation between genetic distance and geographic distance when corrected for salinity (Table 6).

**Table 5:** Results from two AMOVAs examining the level of variation within and among groups based on sample sites. Hierarchical population groupings are in Table 1

Source of variation	Degrees of freedom	Percentage of variation
(1) All populations		
Among groups	4	3.36
Among populations within groups	8	1.11
Within populations	1621	95.54
(2) Estuary populations excluded		
Among groups	3	0.85
Among populations within groups	6	0.52
Within populations	1342	98.64

**Table 6:** Results from simple and partial mantel tests comparing genetic differentiation (*F*_ST_), geographic distance, and salinity between populations. Values in bold are significant (*P* < 0.05)

Comparison	Correlation (*r*)	*P*-value
Simple Mantel tests		
Genetic vs. geographic (all)	**0.578**	**0.004**
Genetic vs. geographic (no lagoon)	0.088	0.321
Genetic vs. salinity (all)	**0.754**	**0.003**
Partial Mantel test		
Genetic vs. geographic (salinity corrected)	0.300	0.072
Genetic vs. salinity (geographic corrected)	**0.639**	**0.016**

BARRIER analysis identified the most significant barrier to gene flow in the Chignik system between Bear Creek in the Chignik River and Metrofania Creek in Chignik Lagoon, the two most proximate Freshwater and Estuary collections (Fig. 2). This separation, corresponding to the transition zone between fresh and saltwater, was supported by 96/100 bootstrap samples. No other barriers were supported by >50% of bootstraps.

## Discussion

Genetic structure of Dolly Varden within the Freshwater sampling sites generally mirrors that of sockeye salmon, with Black Lake, Chignik Lake, and the Chignik River forming discrete groups (Templin et al. 1999; Creelman et al. 2011). However, there were three deviations from the expected geographic pattern of clustering within freshwater for Dolly Varden. Geographically, Bearskin Creek was expected to group with Chignik Lake, as it is a tributary of the lake. However, historic maps (U.S. Geological Survey, 1963) of the Chignik watershed indicate that Bearskin was formerly a tributary of the West Fork River. Channel migrations have occurred over the last several decades and Bearskin Creek now drains directly into Chignik Lake, highlighting the importance of historical geomorphology when interpreting contemporary population structure (Garvin et al. 2013). Second, Hatchery Point Creek clusters between Black Lake, Chignik Lake and Westfork populations, although it drains directly into the middle of Chignik Lake. Hatchery Point Creek appears to be somewhat anomalous, as it was the only stream where fish were found in one of two sampling years, and may have only sporadic use by Dolly Varden. The stream bed is a high gradient scree field that may scour redds during high winter and spring flows, reducing embryo survival. Third, Dolly Varden in Chiaktuak Creek clustered with Black Lake (Alec River) collections but sockeye salmon from Chiaktuak Creek clustered with Chignik Lake, a difference which may be driven by run timing. Specifically, sockeye salmon in Chignik Lake and Chiaktuak Creek tributaries share a similar late spawn timing (Creelman et al. 2011), and straying between the two systems may be successful. However, the close proximity of Black Lake and Chiaktuak Creek may determine Dolly Varden straying rates more than differences in spawn timing.

Initially we hypothesized an isolation by distance model to explain patterns of population structure, where philopatry produces increasing reproductive isolation with increasing distance among spawning habitats. Although we observed isolation by distance when all collections were included but most of the relationship was driven by the Estuary streams clustering geographically at one end of the watershed. Populations within the Chignik system itself did not show the predicted isolation by distance pattern. Similarly, no significant isolation by distance was observed in Chignik Lakes sockeye salmon when only neutral markers were analyzed (Creelman et al. 2011). In the absence of a physical barrier to gene flow, three mechanisms may contribute to the apparent barrier to gene flow detected between Freshwater and Estuary populations: (1) secondary contact between genetic lineages isolated during Pleistocene glaciations; (2) genetic drift in Dolly Varden populations inhabiting smaller Estuarine streams; or (3) different selection regimes among Freshwater and Estuarine stream habitats. Here, we argue that selection for alternative life histories is the most plausible explanation for the observed differences.

The Alaska Peninsula is a zone of secondary contact for other species isolated to the north and south during the last glacial maximum (e.g., chum salmon, Petrou et al. 2013). Dolly Varden occur as two subspecies in Alaska, northern form *S. m. malma*, and southern form *S. m. lordi* (Mecklenburg et al. 2002). The northern form is thought to be distributed from the Mackenzie River to the Alaska Peninsula, and the southern form from the Alaska Peninsula south; however, the exact ranges and regions where the subspecies come into contact is not known (Behnke 2002; Mecklenburg et al. 2002). Our Chignik collections demonstrated substantial allele frequency differences compared to Dolly Varden collections ranging from the North Slope of Alaska to Bristol Bay (Crane et al. 2005a,b2005b; P. A. Crane, unpublished data), suggesting the Chignik Lakes region is not a contact area for the two forms. Lastly, Lindsey and McPhail (1986) suggest the possibility that Black Lake may at one time have drained north into Bristol Bay. However, genetic data show that Chignik Lakes sockeye salmon are more similar to other collections of sockeye salmon from the South Alaska peninsula than to the collections from the north Alaska Peninsula, and do not indicate that Chignik Lakes sockeye are founded via stream capture from north Peninsula streams (T. Dann, Alaska Department of Fish and Game, Anchorage, personal communication).

The gene flow barrier we observed between Freshwater and Estuary collections could be due to founder effects and genetic drift in small populations in the Estuary. However, these effects should be observable as a reduction in heterozygosity and allelic richness, and with the possible exception of Indian Creek, neither were observed in Estuary collections. In addition, some Freshwater collections were from small streams with restricted spawning habitat similar to Estuary streams, yet none of the Freshwater collections was as divergent at neutral loci as the Estuary streams. Since we were unable to confidently estimate the effective population size with fry collections and sibling removal (not shown), we used the number of sibling groups per 100 fish as a proxy for population size. This analysis revealed that several of the Freshwater streams and Estuary tributaries had similar sibling encounter rates, but the Freshwater populations did not show high levels of neutral genetic differentiation from nearby streams. Small Estuary and Freshwater streams are therefore both likely to have few spawning adults. However, even small numbers of spawners straying from Freshwater to Estuary streams would quickly erode the barrier to gene flow we observed between the two regions. It is therefore unlikely that the differentiation observed between Freshwater and Estuary populations is primarily caused by genetic drift or founder effects. Instead, these data are consistent with reproductive isolation facilitated by selection for alternate juvenile life histories in the Freshwater and Estuary habitats.

Much of the isolation we observed is associated with proximity of spawning habitat to saltwater rather than geographic distance. This was unexpected given that the lagoon is the primary summer rearing habitat for fish originating from both Freshwater and Estuary streams; there is no physical limitation preventing dispersal from Freshwater to Estuary streams or vice versa. Despite the proximity of subadult rearing habitats, the *F*_ST_s between Estuary and Freshwater tributaries in Chignik were much greater than other estimates of *F*_ST_ for Dolly Varden from rivers that may be separated by several watersheds, and 100's of km of marine waters, such as rivers of Alaska's North Slope evaluated with similar techniques (Everett et al. 1997; Crane et al. 2005b). Therefore, the genetic isolation between Freshwater and Estuary spawning sites may come from selection for alternative juvenile life histories in Freshwater and Estuary streams, as larger juveniles (>115 mm FL) were unexpectedly scarce in Estuary streams, indicating that such juveniles are rearing elsewhere. Alternatively, Estuary streams may serve as population sinks, where offspring survival is low for Freshwater spawners that stray into them. However, the genetic analysis do not support the founder effects that would be observed in this scenario. The small size of many of the Estuary tributaries (ca. < 1 m^3^/sec) during summer sampling indicates that there may not be suitable overwinter habitat in these streams, and rearing time in freshwater may be reduced. Poor environmental conditions (e.g., anchor ice, low flow, low productivity) in winter in these streams may compel young Dolly Varden to leave Estuary streams in search of more tolerable habitat, possibly ascending Chignik River, a migration which would require travel through waters of salinity ranging 6–30 ‰. The ability for small Dolly Varden to survive in seawater is unstudied, but in closely related Arctic char *Salvelinus alpinus*, osmoregulatory capacity of juveniles varies by population (Dempson 1993; Nilssen and Gulseth 1998; Jensen and Rikardsen 2008). In general, small char (<120 mm FL) have poor survival in salinities >20‰, particularly as water temperature approaches 0°C, and this may be linked to the generally large size of smolts throughout the *Salvelinus* genus (McCormick and Naiman 1984; Yamamoto and Morita 2002). In our study area, we found no individuals >115 mm FL in Estuary streams, indicating that parr from these streams enter marine waters at a smaller than expected size, and are likely physiologically prepared to do so. If Dolly Varden originating in Freshwater streams produce offspring that cannot smolt at such small body sizes, this life-history difference would limit the survival of progeny of Freshwater strays spawning in Estuary habitats and contribute to genetic isolation.

The potential for markedly different juvenile life histories of Estuary spawned fish is significant because although some work has assessed the marine habitat use by adult Dolly Varden (Morita et al. 2009; Bond and Quinn 2013), much of the ecology of these fish in saltwater remains unknown, especially for juveniles. Otolith chemical analysis of spawning individuals in Estuary streams to verify differences in juvenile life histories would support our hypothesis of early life-history differences between Estuary and Freshwater spawned fish as the mechanism of isolation between these groups.

The stark divergence in population structure with neutral loci that we observed between Freshwater and Estuary streams is rare for a single morph of a highly migratory species among readily accessible habitats (i.e., low gradient, free of impassable waterfalls). Under these circumstances, other studies have often shown little to no significant variation among collections. Most studies identifying highly divergent populations in anadromous fishes have been conducted on much larger spatial scales (e.g., comparing watersheds), or fine spatial scales where movement is confined by physical barriers (Whiteley et al. 2006; Meeuwig et al. 2010; Warnock et al. 2010), individual morphology (Lin et al. 2008) or spawn timing (Quinn et al. 2000). In the Chignik system, however, reproductive isolation is likely generated by differing selective pressures in juvenile life histories among spatially proximate populations, resulting in isolation by adaptation. We found structure despite the fact that Dolly Varden regularly migrate from the saltwater habitat adjacent to Estuary streams, where summer rearing occurs, to headwater habitats (i.e., the estuary to Black Lake) in a number of hours (Bond and Quinn 2013). Previous efforts have not identified Dolly Varden fry or small parr in saltwater in the Chignik area (Narver and Dahlberg 1965; Bond 2013), although Estuary streams are small enough that juvenile production is undoubtedly limited. Therefore, despite regular sampling in estuarine habitats, small emigrants of Estuary streams may go undetected. In addition, movement of young fish into or through saltwater may not occur until the late fall or early winter when saltwater habitats have not been previously monitored. Analogous population structure and life-history diversity has been observed in Chignik Lakes sockeye salmon (Creelman et al. 2011), where a genetically distinct population of fish spawns in Chignik River, immediately upstream from Chignik lagoon (Simmons et al. 2013). Rather than moving to lakes for one or more years of rearing, emergent sockeye fry from the Chignik River move downstream and are tolerant of saltwater at a small body size.

The Chignik watershed is an apparent hotspot for within species diversity, possibly as a zone of contact among divergent lineages. Extensive life history, morphological, or genetic diversity has been demonstrated in several species (Narver 1969; McCart 1970; Gowell et al. 2012; Taugbol et al. 2014). Although this diversity may be the result of postglacial colonization of distinct lineages, the region is extremely volcanically active, and may have been recolonized multiple times following more recent volcanic events (Miller and Smith 1987). Much of the freshwater habitat of southern Alaska Peninsula is comprised of small coastal streams; Chignik Lakes and the Aniakchak River (Surprise Lake) are the only substantial lake bearing streams that drain southward to the Gulf of Alaska. The genetic connectivity or life-history similarity of Chignik Estuary streams with other small coastal drainages remains largely unknown, as most remain uncharacterized. However, Indian Creek, a nearby drainage in Chignik Bay, grouped closely with other Estuary streams, suggesting that other small coastal watersheds may show similar patterns. Future work, therefore should look for similar genetic and life-history characteristics further from the Chignik watershed.

This research highlights the importance of identifying both genetic and life-history diversity at appropriate spatial scales for management. Regional management of anadromous fishes is often driven by a single large stock, or an assumed metapopulation of stocks (Schtickzelle and Quinn 2007). Identifying the spatial extent of genetic population structure is a key goal of management and conservation (Fullerton et al. 2011). However, in addition to analyses that recognize magnitude of genetic exchange among connected habitats, genetic analysis can be employed to detect the presence and spatial extent of previously unidentified morphs or life histories by identifying regions of genetic discontinuity among proximate habitats. This is particularly useful in fishes, where direct contact with all life stages may not be feasible. Genetics can therefore identify likely locations to focus further research efforts. Although other, less likely scenarios may produce the observed patterns, we suggest that the presence of a novel Dolly Varden life history from a combination of genetic and juvenile size-distribution data result from isolation by adaptation on a fine spatial scale. Therefore, Dolly Varden in the Chignik Lakes region comprised a large group of phenotypically and genotypically similar Freshwater spawning individuals, and a distinct group of many small streams composed of Estuary fish, although the full life history of Estuary fish remains unknown. Therefore, more work is needed to identify both the spatial extent and full life history of the Estuary population, as many small unassessed coastal streams throughout southwestern Alaska may contain Dolly Varden with similar life histories. In addition, there may be interactions between Estuary and Freshwater fish during juvenile rearing or overwintering of adults as Estuary habitats are unsuitable for extensive rearing. Therefore, further exploration of habitat use by Estuary born fish through mixed stock analysis of fish found in marine and lacustrine rearing habitats, or otolith chemical analysis of Estuary spawners is warranted.

## Figures and Tables

**Figure A1:**
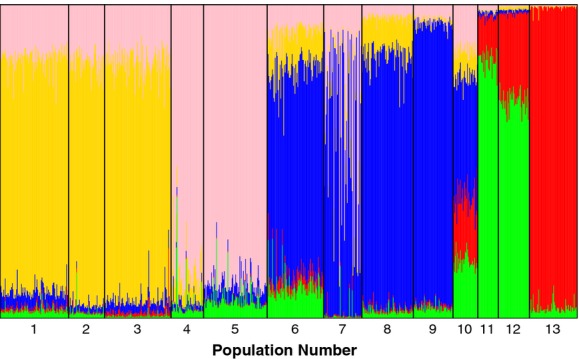
Results from STRUCTURE clustering analysis for two data sets all 13 populations sampled in the Chignik system (*K* = 5). Populations are ordered to reflect the geography of the Chignik system and population numbers are found in Table 1.

## Appendix A2

**Table A2A:** *P*-values for exact tests of genetic differentiation conducted in ARLEQUIN 3.5

	Boul	Alec	Chia	Cloud	Bskin	Cucu	Hatc	Fonz	Disa	Bear	Metro	Spit
Alec	0.726											
Chia	0.002	0.054										
Cloud	0.000	0.024	0.000									
Bskin	0.000	0.000	0.000	0.003								
Cucu	0.000	0.000	0.000	0.000	0.000							
Hatc	0.001	0.010	0.000	0.010	0.000	0.000						
Fonz	0.000	0.000	0.000	0.000	0.000	0.001	0.000					
Disa	0.000	0.000	0.000	0.000	0.000	0.015	0.000	0.000				
Bear	0.000	0.000	0.000	0.000	0.000	0.001	0.000	0.000	0.000			
Metro	0.000	0.000	0.000	0.000	0.000	0.000	0.000	0.000	0.000	0.000		
Spit	0.000	0.000	0.000	0.000	0.000	0.000	0.000	0.000	0.000	0.000	0.000	
Indi	0.000	0.000	0.000	0.000	0.000	0.000	0.000	0.000	0.000	0.000	0.000	0.000

**Table A2B:** Evanno table output for STRUCTURE run with all populations (Fig. 3A). The most likely value of *K* based on the delta *K* method (*K* = 2) is highlighted in gray. See Evanno et al. (2005) and Earl and vonHoldt 2012 (2012) for further information on this type of table

*K*	Reps	Mean LnP (*K*)	Stdev LnP (*K*)	Ln’(*K*)	|Ln’’(*K*)|	Delta *K*
1	10	−35886.47	0.18	–	–	–
2	10	−34936.10	24.22	950.37	671.63	27.7253
3	10	−34657.36	18.23	278.74	137.73	7.5566
4	10	−34516.35	47.14	141.01	42.57	0.9030
5	10	−34332.77	58.89	183.58	283.66	4.8169
6	10	−34432.85	138.23	−100.08	32.35	0.2340
7	10	−34500.58	117.54	−67.73	118.79	1.0106
8	10	−34687.10	257.05	−186.52	3.67	0.0143
9	10	−34869.95	478.70	−182.85	176.65	0.3690
10	10	−34876.15	372.62	−6.20	–	–

**Table A2C:** Evanno table output for STRUCTURE run with only the Freshwater populations (Fig. 3A). The most likely value of *K* based on the delta *K* method (*K* = 3), is highlighted in gray. See Evanno et al. (2005) and Earl and vonHoldt 2012 (2012) for further information on this type of table

*K*	Reps	Mean LnP (*K*)	Stdev LnP (*K*)	Ln’(*K*)	|Ln’’(*K*)|	Delta *K*
1	10	−29285.65	0.37	–	–	–
2	10	−29047.45	80.86	238.20	26.77	0.3311
3	10	−28836.02	30.06	211.43	453.04	15.0731
4	10	−29077.63	153.90	−241.61	115.70	0.7518
5	10	−29203.54	291.99	−125.91	180.95	0.6197
6	10	−29148.50	164.83	55.04	167.61	1.0168
7	10	−29261.07	277.95	−112.57	13.63	0.0490
8	10	−29360.01	408.06	−98.94	253.00	0.6200
9	10	−29711.95	380.62	−351.94	657.16	1.7265
10	10	−29406.73	297.67	305.22	–	–
